# Comparative effectiveness of anti-inflammatory and antiviral therapies on NLR and survival outcomes in severe COVID-19: multicenter retrospective study

**DOI:** 10.7717/peerj.20003

**Published:** 2025-09-04

**Authors:** Abbas Al Mutair, Muhammad Daniyal, Sameer A. Alkubati, Hamdan Albaqawi, Awatif M. Alrasheeday, Bushra Alshammari, Kawthar Alsaleh, Richard Mottershead, Hanan Alyami, Hanan F. Alharbi, Awad Al-Omari

**Affiliations:** 1 Research Center, Almoosa Specialist Hospital, Al-ahsa, Saudi Arabia; 2 College of Nursing, Princess Norah Bint Abdulrahman University, Riyadh, Saudi Arabia; 3 Department of Nursing, Prince Sultan Military College of Health Sciences, Dahran, Saudi Arabia; 4 School of Nursing, University of Wollongong, Sydney, Australia; 5 Department of Nursing, Almoosa College of Health Science, All-ahsa, Saudi Arabia; 6 Medical Surgical Nursing Department, University of Hail, Hail, Saudi Arabia; 7 Nursing Administration Department, University of Hail, Hail, Saudi Arabia; 8 Department of Nursing, College of Health Sciences, University of Sharjah, Sharjah, United Arab Emirates; 9 Department of Maternity and Pediatric Nursing, College of Nursing, Princess Nourah bint Abdulrahman University, Riyadh, Saudi Arabia; 10 Almana Health Group, Al-Khobar, Saudi Arabia

**Keywords:** Neutrophil-to-Lymphocyte ratio, Antiviral drugs, Immune response, Inflammation biomarker, Respiratory illness, Critical care, Illness

## Abstract

**Background:**

The COVID-19 pandemic has highlighted the critical role of immune dysregulation and systemic inflammation in disease severity, particularly in patients with severe respiratory illness. Elevated levels of pro-inflammatory cytokines, such as IL-6, and biomarkers like the neutrophil-to-lymphocyte ratio (NLR) have been associated with worse outcomes. This study enrolled laboratory-confirmed SARS-CoV-2 patients with acute respiratory illness requiring intestive care unit (ICU) admission, including mechanical ventilation, to evaluate the effect of different treatments on NLR, neutrophil count (NC), and lymphocyte count (LC).

**Methods:**

A retrospective, multicenter, observational cohort study was conducted across 15 tertiary hospitals in Saudi Arabia, involving 1,490 ICU-admitted COVID-19 patients between March 1, 2020, and October 30, 2020. Data on patient demographics, comorbidities, laboratory results, and treatment outcomes were collected using the Research Electronic Data Capture (REDCap) system. The study evaluated the effect of different treatments on NLR, neutrophil count (NC), and lymphocyte count (LC).

**Results:**

This study utilized 1,490 patients in the study of whom 73.6% were male and 26.1% were female. The average age of patients was 56.2 years, with a mean NLR of 8.77 ± 8.64, showing significant systemic inflammation. Tocilizumab (*p* = 0.031), oseltamivir (*p* = 0.004), and linezolid (0.029) showed statistically significant effects on NLR. Tocilizumab demonstrated the highest mean survival time with 60.813 days, compared to linezolid (49.359 days) and ostilomavir (40.635 days). However, patients not getting linezolid or ostilomavir had longer mean survival times, suggesting potential limitations in their efficacy. Tocilizumab also showed a weak positive correlation with NC (*r* = 0.086, *p* = 0.001), further supporting its role in modulating inflammation and improving the immune system.

**Conclusion:**

Among the evaluated therapies, tocilizumab and oseltamivir showed a consistent trend of lower NLR values in both survivors and non-survivors, compared to those not receiving these treatments. These findings suggest that tocilizumab and oseltamivir may offer some efficacy in modulating immune response (as measured by NLR) and potentially improving outcomes. However, due to observed weak correlations no single therapy alone appears sufficient to predict or reduce mortality, emphasizing the need for multimodal treatment strategies and further investigation into combined biomarker models.

## Introduction

Notable epidemics consist of severe acute respiratory syndrome (SARS) in 2003 and the Middle East respiratory syndrome (MERS), which began in 2012. Major pandemics include the H1N1 influenza and the coronavirus disease 2019 (COVID-19) caused by SARS-CoV-2. These infections can cause serious problems and affect normal breathing. They can cause flu (influenza), severe acute respiratory syndrome (SARS), and COVID-19. The COVID-19 pandemic has profoundly affected global health as the severe respiratory virus (Zhou et al., 2020; Coronaviridae Study Group of the International Committee on Taxonomy of Viruses, 2020). The neutrophil-to-lymphocyte ratio (NLR) is a measure of systemic inflammation derived from the white blood cell (WBC) count, one of the most common infection markers. It has been used as a predictor of cardiovascular diseases (Azab et al., 2010) and cancer (Walsh et al., 2005). Zahorec (2001) proposed the use of the NLR as an additional infection marker in clinical intensive care unit practice based on the phenomenon that the physiological immune response of circulating leukocytes to various stressful events is often characterized by an increase in neutrophil counts and a decline in lymphocyte counts. Additionally, according to acute physiology and APACHE II and SOFA scores, it was found that NLR correlated well with the severity of disease and outcome (Zhou et al., 2019). Studies have shown that it is a systemic inflammatory marker used as a determinant of the diagnosis as well as the prognosis of patients with a viral or bacterial infection (Li et al., 2021; Önal et al., 2022). COVID-19 is marked by elevated plasma levels of pro-inflammatory cytokines, ferritin, lactate dehydrogenase (LDH), D-dimer, and C-reactive protein, indicating systemic inflammation (Teachey et al., 2013; Moore & June, 2020; Mehta et al., 2020). Lung tissue histopathology in deceased patients has revealed inflammatory cellular infiltration, proteinaceous exudate, and extensive alveolar edema, emphasizing pulmonary inflammation and cytokine storm resulting from immune dysregulation (Teachey et al., 2013; Moore & June, 2020). Tocilizumab, a monoclonal antibody targeting the pro-inflammatory cytokine IL-6, has been approved for the treatment of cytokine release syndrome (CRS) (Teachey et al., 2013). It has demonstrated rapid improvement in respiratory and hemodynamic parameters in CRS, and the US Food and Drug Administration has endorsed its use for severe or life-threatening CRS (Guaraldi et al., 2020). Severe respiratory illnesses, including viral infections such as COVID-19, often lead to a hyper-inflammatory state, which can result in respiratory failure and poor patient outcomes. In these cases, immune dysregulation plays an essential role, with an elevation in pro-inflammatory cytokines such as IL-6, TNF-α, and IL-1 contributing to systemic inflammation that worsens illness severity (Qin et al., 2020; Tomita et al., 2011). NLR can mirror the derangement between innate and adaptive immunity in several pathophysiological states, including neoplastic diseases, and not only in inflammatory diseases (Buonacera et al., 2022). High NLR is particularly relevant in diseases like COVID-19, where it has been demonstrated that an increased NLR serves as an early predictor of severe disease progression and complications (Forget et al., 2017; Tomita et al., 2011; Lee et al., 2010; Azab et al., 2012; Proctor et al., 2011; Koza, 2016; Hao et al., 2017; De Jager et al., 2012; Terradas et al., 2012; Bozbay et al., 2014). It is important to specify that the data reported by De Jager et al. (2012) on the NLR were obtained from patients with community acquired pneumonia (CAP). This finding was further confirmed in older patient populations by Cataudella et al. (2017), suggesting that the prognostic value of NLR in CAP patients is independent of aging. Numerous studies have confirmed that elevated NLR correlates with poor clinical outcomes and can predict the severity of respiratory illnesses, making it a valuable tool for clinicians to monitor patient status and identify those at higher risk for complications (Liu et al., 2020b; Liu et al., 2020a; Chan & Rout, 2020; Feng et al., 2020; Fu et al., 2020; Kerboua, 2020; Kong et al., 2020; Lagunas-Rangel, 2020; Lian, 2020; Tatum et al., 2020; Wang et al., 2020; Calvin, Wijaya & Ibrahim, 2021).

This study will evaluate the relationship between immune response markers, specifically NLR, NC, and LR, and the efficacy across several different treatments in patients with severe respiratory illnesses, using multi-center data of COVID-19 patients as a special case. The NLR, along with absolute neutrophil (N) and lymphocyte (L) counts, was selected as primary biomarkers due to their well-documented role in quantifying immune dysregulation and systemic inflammation. Neutrophils drive pro-inflammatory responses through cytokine release and oxidative burst, while lymphocytes mediate adaptive immunity and regulatory functions (Tomita et al., 2011; Forget et al., 2017; Lee et al., 2010; Azab et al., 2012; Proctor et al., 2011).

This large, multicenter study involving 1,490 critically ill COVID-19 patients offers a unique opportunity to evaluate immune and inflammatory responses within a relatively uniform disease model. Unlike conditions such as sepsis, unlike conditions such as sepsis, which involve diverse pathogens and varied immune reactions. While sepsis can also trigger cytokine storms and immune dysregulation (Regolo et al., 2023; Chaudhry et al., 2013; Jarczak & Nierhaus, 2022), COVID-19 exhibits a more stereotyped hyperinflammation pattern driven predominantly by SARS-CoV-2-specific mechanisms (*e.g.*, IL-6/IL-1β dominance, endothelial injury). This relative uniformity enhances the clarity of biomarker–treatment relationships in COVID-19. By integrating these markers as predictors of treatment outcomes, this study will provide insights into how these immune parameters correlate with different treatment efficacy.

## Materials & Methods

### Design and setting of the study

This is a retrospective, multicenter, observational cohort study from 15 tertiary public and private hospitals located in different geographical areas across the Kingdom of Saudi Arabia. The informed consent was obtained from each individual participating in the study on an electronic consent form. This study included laboratory-confirmed SARS-CoV-2 patients as the special case study of acute respiratory illness admitted to the intensive care units across Saudi Arabia between March 1, 2020, and October 30, 2020. A total of 1,490 critically ill SARS-CoV-2 patients were included. The study was powered based on the effect size from a previous study (Qin et al., 2020), with a 99% confidence interval and a small margin of error (*e* = 0.01), using z-statistics of 2.85. All participants were severe COVID-19 patients on mechanical ventilation, treated with standard care.

For each patient, the NLR was calculated using laboratory results recorded within the first 24 h of intensive care unit (ICU) admission (designated Day 1). These values were used as baseline indicators of systemic inflammation and were analyzed with the administered treatments to assess initial immune modulation. Longitudinal trends in NLR were not evaluated in this study. The start time for observation was the date of ICU admission (recorded as “Day 1” for each patient, per screening criteria of the study). Outcome data (including NLR, mortality, and survival time) were collected throughout the ICU/hospital stay until discharge, death, or study end (October 30, 2020).

### Inclusion and exclusion criteria

This multicenter cohort study enrolled adult patients with laboratory-confirmed SARS-CoV-2 infection through validated testing methods (nasopharyngeal swab, sputum/tracheal aspirate, or BAL) who were admitted to ICUs between March 1 and October 30, 2020. The study specifically targeted critically ill COVID-19 cases requiring intensive care, as evidenced by mechanical ventilation needs or severe respiratory distress. Key inclusion requirements comprised complete demographic data, documented inflammatory markers (particularly NLR values), and proper informed consent. Exclusion criteria eliminated non-ICU patients, those with incomplete clinical/laboratory records, asymptomatic/mild cases, and individuals hospitalized outside the study window. This ensured analysis focused exclusively on severe COVID-19 presentations with comprehensive treatment and outcome data. All enrolled patients had severe COVID-19 illness, operationally defined as requiring ICU admission and mechanical ventilation. This ensured a homogeneous population of critically ill patients for assessing the relationship between inflammatory markers and treatment outcomes. 

### Ethical approval

Ethical approval for this study was obtained from the Central Institutional Review Board at the Saudi Ministry of Health (IRB Registration No. H-01-R-009) and the Institutional Review Board registered with the King Abdulaziz City for Science and Technology (KACST), Saudi Arabia (IRB Registration No. H-01-R-012). All methods were performed according to the relevant guidelines and regulations. Data was collected using the Research Electronic Data Capture (REDCap) system, a secure online platform for data management and analysis. The Central Institutional Review Board approved the study at the Saudi Ministry of Health and the ethics boards of participating centers.

### Variables of the study under investigation

The variables in the study were categorized as follows:

Demographic variables: age, sex, BMI, smoking status, and comorbidities (*e.g.*, diabetes, hypertension, cardiovascular disease, chronic kidney disease, chronic lung disease). Inflammatory and immune markers: NLR, neutrophil count, and lymphocyte count, measured at ICU admission (Day 1). Therapeutic interventions: administration of antivirals (*e.g.*, remdesivir, oseltamivir), biological agents (*e.g.*, tocilizumab, IVIG), and (*e.g.*, linezolid (used for suspected or confirmed Gram-positive bacterial co-infections), vancomycin), categorized as binary (received/not received). Outcomes: ICU mortality, hospital mortality, ICU length of stay, hospital length of stay, and survival duration (in days). Secondary bacterial infections were identified clinically and managed per institutional protocols. Although microbiological confirmation was not consistently available across centers, the use of antibiotics such as linezolid in selected cases reflects clinical suspicion or diagnosis of co-infection.

The study explicitly states that NLR values were calculated using laboratory results recorded within the first 24 h of ICU admission (designated as Day 1). These baseline values served as indicators of systemic inflammation at the onset of critical illness. The primary aim was to evaluate the association between baseline NLR and treatment efficacy, not to track dynamic changes over time. The NLR at admission was used as a predictor of treatment outcomes (*e.g.*, survival, mortality) rather than as a longitudinal marker of treatment response.

### Statistical analysis

For continuous variables such as age, body mass index (BMI), and laboratory values average with their SDs will be computed. Descriptive statistics, such as frequencies and percentages, were used to display categorical variables. Inferential statistics will be applied to measure the significance and testing of continuous and qualitative variables. While for categorical variables the *χ*2-test (or Fisher’s exact test for 2 × 2 categories) will be applied to compare the proportions between the two groups. The normality of the continuous variables has been tested by the Shapiro–Wilk test. Multivariate analysis of variance by the GLM model will be applied to measure the effect of different treatments on the NLR ratios. All data analyses will be performed using IBM SPSS Statistics software version 27.0 (IBM Corp., Armonk, NY, USA) and R-Studio with results considered statistically significant at a two-sided *p*-value of ≤ 0.05.

## Results

**Figure 1:**
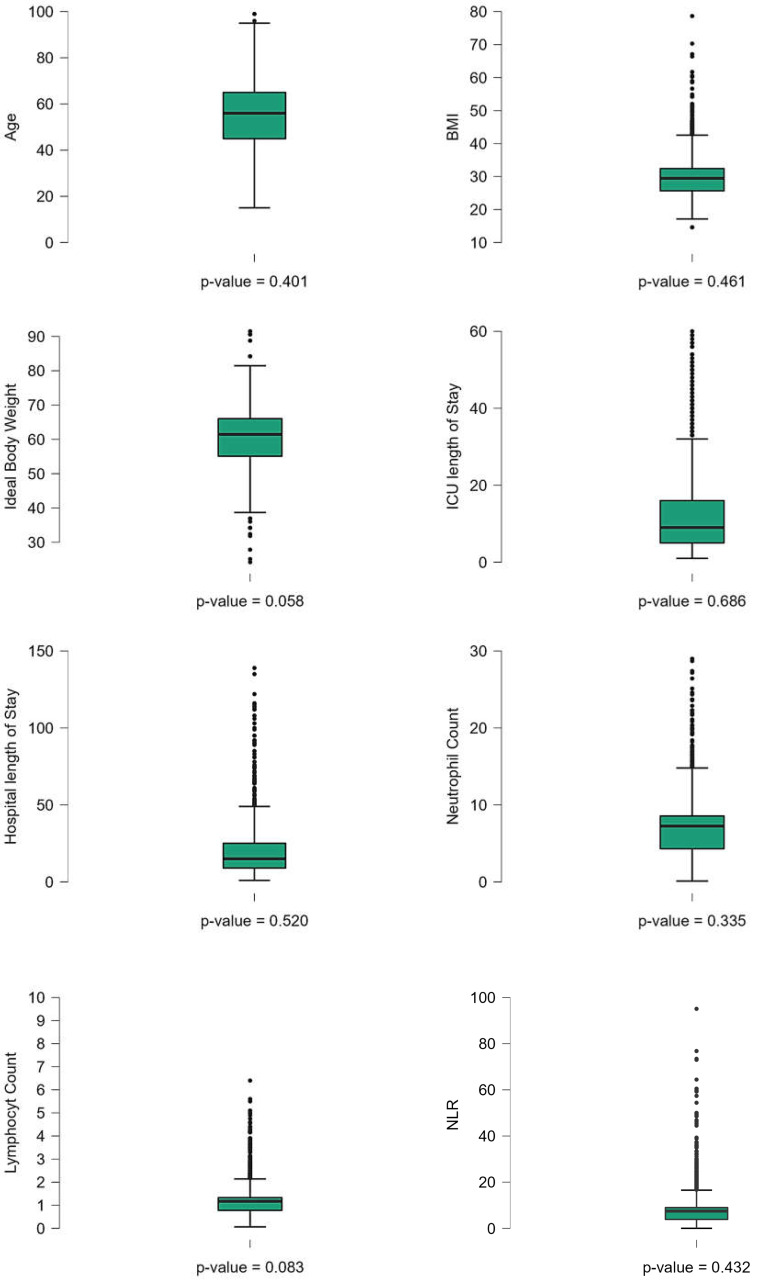
Boxplots of key clinical and baseline characteristics of COVID-19 patients. Boxplots illustrating the distribution of key clinical and baseline characteristics among COVID-19 patients. Differences were considered statistically significant at *p* < 0.05.

**Figure 2:**
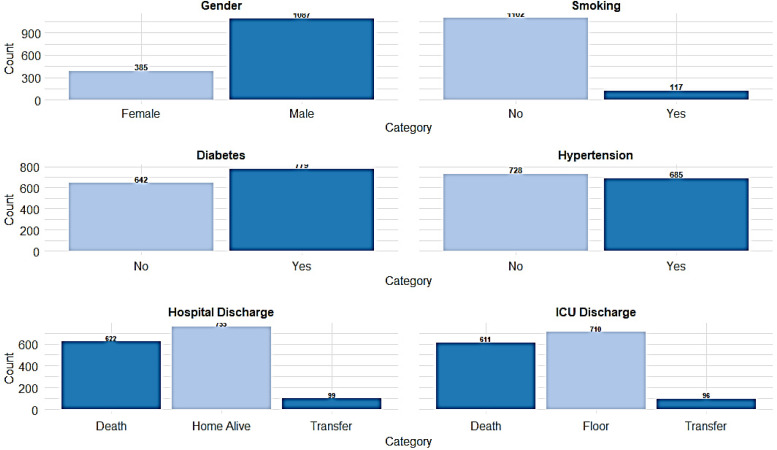
The distribution of key categorical variables among the patient population.

The study involved 1,490 ICU-admitted COVID-19 patients, serving as a detailed case study of acute respiratory illness. The mean age of the patients was 56.2 ± 15.7 years. The average length of stay in the ICU was 13.2 ± 14.6 days, while the total hospital length of stay averaged 21.2 ± 19.3 days. Regarding body composition, the mean ideal body weight was 60.47 ± 9.17 kg, and the mean body mass index (BMI) was 30.13 ± 6.55 kg/m. Hematological parameters showed that the mean white blood cell count was 9.62 ± 7.05 ×10^9^/L, with a mean lymphocyte count of 1.34 ± 4.35 ×10^9^/L and a mean neutrophil count of 7.26 ± 4.42 ×10^9^/L. The NLR, a critical marker of systemic inflammation, had a mean value of 8.77 ± 8.64. The normality of the clinical continuous variables has been evaluated and presented (Table 1, Fig. 1). Among the study population, males comprised the majority, with 1,087 (73.6%), while females accounted for 385 (26.1%). Travel history was documented for 122 (8.3%) reporting recent travel, while 1,021 (69.2%) had no travel history, and 333 (22.6%) had missing data. Close contact exposure was confirmed in 434 (29.4%) patients, while 572 (38.8%) had no known exposure, and 470 (31.8%) had undocumented exposure status. A total of 151 (10.2%) patients reported attending large gatherings before contracting COVID-19, while 749 (50.7%) denied such exposure, and 576 (39.0%) had no documented information. Nosocomial infection was identified in 170 (11.5%) cases, whereas 944 (64.0%) had no hospital-acquired infection, and data were missing for 362 (24.5%) patients. Notably, unknown exposure sources were common, with 831 (56.3%) patients reporting an unidentified origin of infection. Regarding comorbidities, diabetes was prevalent in 779 (52.8%) patients, while 642 (43.5%) were non-diabetic, and 55 (3.7%) had undocumented diabetes status. Hypertension was diagnosed in 685 (46.4%) patients, whereas 728 (49.3%) were non-hypertensive, with missing data in 63 (4.3%) cases. Cardiovascular diseases were also noted, with 215 (14.6%) patients having ischemic heart disease, 108 (7.3%) reporting heart failure, and 156 (10.6%) diagnosed with chronic kidney disease. Additionally, 94 (6.4%) patients were on dialysis, and 72 (4.9%) had a history of organ transplantation. Smoking was reported in 117 (7.9%) patients, while 1,102 (74.7%) were non-smokers (Fig. 2). Chronic lung conditions were observed in 78 (5.3%) patients, with 65 (4.4%) diagnosed with chronic obstructive pulmonary disease (COPD) and 167 (11.3%) having asthma. Cancer was identified in 89 (6.0%) patients, while 76 (5.1%) had undergone recent surgery. In terms of hospitalization outcomes, 755 (51.2%) patients were discharged home alive, while 622 (42.1%) died, and 99 (6.7%) were transferred to another facility. ICU discharge outcomes revealed that 710 (48.1%) patients were transferred to a general hospital ward, while 611 (41.4%) died, 96 (6.5%) were transferred to another facility, and 59 (4.0%) were discharged home (Table 1, Fig. 2).

**Table 1:** Baseline and clinical characteristics of patients in the study (*N* = 1,490).

**Variable of the study**	**Measures**	
Age	56.2 ± 15.7	
ICU length of stay	13.2 ± 14.6	
Hospital length of stay	21.2 ± 19.3	
Ideal body weight	60.47 ± 9.17	
Body mass index	30.13 ± 6.55	
White blood cell count	9.62 ± 7.05	
Lymphocyte count	1.34 ± 4.35	
Neutrophil count	7.26 ± 4.42	
Neutrophil to lymphocyte ratio	8.77 ± 8.64	

	**Category**	**Count (n) with percentage**
Gender	Male	1,087 (73.6%)
	Female	385 (26.1%)
Travel history	Yes	122 (8.3%)
	No	1,021 (69.2%)
	Not documented	333 (22.6%)
Close contact exposure	Yes	434 (29.4%)
	No	572 (38.8%)
	Not documented	470 (31.8%)
Large gathering attendance	Yes	151 (10.2%)
	No	749 (50.7%)
	Not documented	576 (39.0%)
Nosocomial infection	Yes	170 (11.5%)
	No	944 (64.0%)
	Not documented	362 (24.5%)
Unknown exposure source	Yes	831 (56.3%)
	No	417 (28.3%)
	Not documented	228 (15.4%)
Smoking status	Yes	117 (7.9%)
	No	1,102 (74.7%)
	Not documented	257 (17.4%)
Diabetes	Yes	779 (52.8%)
	No	642 (43.5%)
	Not documented	55 (3.7%)
Hypertension	Yes	685 (46.4%)
	No	728 (49.3%)
	Not documented	63 (4.3%)
Ischemic heart disease	Yes	215 (14.6%)
	No	1,157 (78.4%)
	Not documented	104 (7.0%)
Heart failure	Yes	108 (7.3%)
	No	1,265 (85.7%)
	Not documented	103 (7.0%)
Chronic lung disease	Yes	78 (5.3%)
	No	1,293 (87.6%)
	Not documented	105 (7.1%)
COPD	Yes	65 (4.4%)
	No	1,305 (88.4%)
	Not documented	106 (7.2%)
Asthma	Yes	167 (11.3%)
	No	1,211 (82.0%)
	Not documented	98 (6.6%)
Chronic liver disease	Yes	64 (4.3%)
	No	1,314 (89.0%)
	Not documented	98 (6.6%)
Hemoglobinopathy	Yes	47 (3.2%)
	No	1,326 (89.8%)
	Not documented	103 (7.0%)
Chronic kidney disease	Yes	156 (10.6%)
	No	1,224 (82.9%)
	Not documented	96 (6.5%)
Dialysis	Yes	94 (6.4%)
	No	1,284 (87.0%)
	Not documented	98 (6.6%)
Organ transplant history	Yes	72 (4.9%)
	No	1,310 (88.8%)
	Not documented	94 (6.4%)
Immunocompromised	Yes	113 (7.7%)
	No	1,265 (85.7%)
	Not documented	98 (6.6%)
Chronic hematologic disease	Yes	54 (3.7%)
	No	1,330 (90.1%)
	Not documented	92 (6.2%)
HIV AIDS	Yes	43 (2.9%)
	No	1,341 (90.9%)
	Not documented	92 (6.2%)
Cancer status	Yes	89 (6.0%)
	No	1,290 (87.4%)
	Not documented	97 (6.6%)
Recent surgery	Yes	76 (5.1%)
	No	1,289 (87.3%)
	Not documented	111 (7.5%)
Hospital discharge	Transfer to another facility	99 (6.7%)
	Discharge home alive	755 (51.2%)
	Death	622 (42.1%)
ICU discharge	Discharged to floor	710 (48.1%)
	Transfer to another facility	96 (6.5%)
	Discharge home	59 (4.0%)
	Death	611 (41.4%)

**Figure 3:**
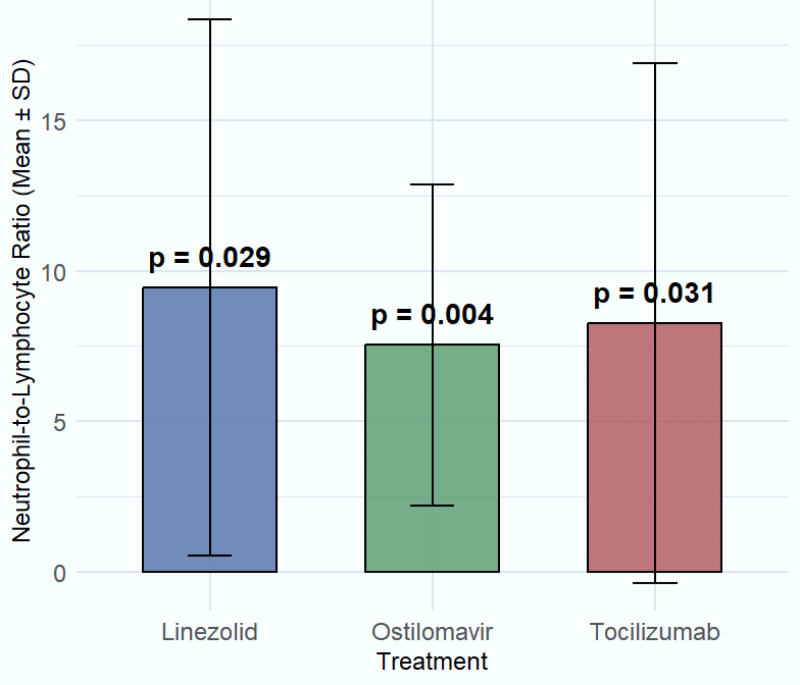
Comparison of significant treatments on NLR. The effects of four significant treatments—linezolid, tocilizumab and oseltamivir on neutrophil-to-lymphocyte ratio (NLR) values.

For estimating the relationship of NLR with various treatments, a generalized linear model has been applied. The study estimated that hydroxychloroquine (8.33 ± 8.94, *p*-value = 0.620), chloroquine (8.86 ± 7.71, *p*-value = 0.762), and azithromycin (8.81 ± 8.84, *p*-value = 0.339) did not significantly impact the NLR, as indicated by *p*-values greater than 0.05. Similarly, Kaletra (8.50 ± 7.89, *p*-value = 0.602) and favipiravir (9.01 ± 9.78, *p*-value = 0.321) showed no significant changes in the NLR, suggesting their limited role in modulating immune responses in COVID-19 patients. The treatment remdesivir (9.82 ± 7.13, *p*-value = 0.426) did not show significant improvement in NLR either, although it is still used for viral suppression. Ribavirin (9.20 ± 9.13, *p*-value = 0.115), and β-Lactam/BLI (8.30 ± 8.24, *p*-value = 0.055) demonstrated modest effects on NLR, with none showing statistical significance. Significant associations with NLR were observed for oseltamivir (7.54 ± 5.33, *p* = 0.004) and linezolid (9.44 ± 8.91, *p* = 0.029), a broad-spectrum antibiotic used for suspected or confirmed Gram-positive bacterial co-infections complicating COVID-19, also demonstrated a statistically significant association with NLR. However, this association likely reflects the presence and severity of the underlying bacterial co-infection or the response to its treatment, rather than a direct immunomodulatory effect on COVID-19 pathophysiology. While these findings indicate a statistical difference in NLR values among patients receiving these treatments, particularly for linezolid, the results should be interpreted carefully. Given that linezolid is typically used in the presence of suspected bacterial co-infection, a factor not systematically stratified in this study, its effect on NLR may reflect underlying infection severity rather than a direct immunomodulatory effect. Similarly, tocilizumab (8.27 ± 8.63, *p* = 0.031) also showed a statistically significant reduction in NLR, further supporting its relevance in modulating immune response. These findings highlight the potential of otilimavir, linezolid, and tocilizumab as valuable treatments for managing immune-related complications in COVID-19, justifying further clinical investigation. In contrast, ceftalazone-avibactam (10.15 ± 10.56, *p* = 0.506) and ceftazidime-tazobactam (9.26 ± 8.27, *p* = 0.667) exhibited higher mean NLR values but did not produce statistically significant changes, suggesting a limited impact on inflammation or immune cell regulation (Table 2, Fig. 3).

Table 2 compares baseline NLR values at ICU admission between patients who received specific treatments and those who did not. Significant differences (*e.g.*, for tocilizumab, oseltamivir, linezolid) suggest these treatments were selectively given to patients with different inflammatory profiles. Notably, this analysis does not evaluate NLR changes during treatment, as NLR was measured only at admission. The study did not monitor longitudinal NLR changes (post-treatment) due to its retrospective design. Baseline NLR (Day 1) was the only NLR value analyzed.

### Relationship of immune response and inflammation levels with treatments

The study from 1,490 patients showed that NLR had a weak but significant positive correlation with ostilomavir (Tamiflu) (*r* = 0.077, *p*-value = 0.004), but no significant correlation with tocilizumab (*r* = 0.037, *p*-value = 0.153) or linezolid (*r* = −0.031, *p*-value = 0.246). In terms of the neutrophil count, it a weak positive correlation with tocilizumab (*r* = 0.086, *p*-value = 0.001), but no significant correlation with linezolid (*r* = −0.009, *p*-value = 0.748) or oseltamivir (Tamiflu) (*r* = 0.023, *p*-value = 0.388). The study noted that for lymphocyte count, there was a weak negative correlation with oseltamivir (Tamiflu) (*r* = −0.053, *p*-value = 0.044). There was no significant correlation with tocilizumab (*r* = 0.024, *p*-value = 0.348) or linezolid (*r* = 0.011, *p*-value = 0.685). Tocilizumab appears to be the treatment that performed relatively better in terms of its statistically significant association with the neutrophil count, which is a critical indicator of inflammatory responses. However, oseltamivir showed some effect on NLR and lymphocyte count as well, but not as strongly. The lack of significant correlation between linezolid and immune markers (NLR, NC, LC) further supports the interpretation that its primary effect relates to managing bacterial co-infection rather than directly modulating the COVID-19 immune response.

**Table 2:** Baseline NLR association with treatment.

**Treatment**	**Lymphocyte_Count_1 (Mean ± SD)**	**Neutrophil_Count_1 (Mean ± SD)**	**Neutrophil-to-Lymphocyte ratio (Mean ± SD)**	**Beta**	***p*-value (NLR)**
Hydroxychloroquine	2.25 ± 0.74	7.88 ± 4.20	8.33 ± 8.94	−0.042	0.620
Chloroquine	2.02 ± 0.59	8.14 ± 4.42	8.86 ± 7.71	−0.013	0.762
Azithromycin	2.36 ± 5.10	8.25 ± 4.52	8.81 ± 8.84	−0.008	0.339
Kaletra	2.26 ± 0.90	8.12 ± 4.24	8.50 ± 7.89	−0.029	0.602
Tocilizumab	2.17 ± 0.63	7.67 ± 4.00	8.27 ± 8.63	−0.081	0.031^**^
Favipiravir	2.25 ± 0.74	7.99 ± 3.88	9.01 ± 9.78	−0.028	0.321
Remdesivir	1.93 ± 0.48	7.96 ± 3.38	9.82 ± 7.13	−0.021	0.426
Ribavirin	2.22 ± 0.88	8.35 ± 4.74	9.20 ± 9.13	−0.066	0.115
IVIG	2.21 ± 0.72	7.97 ± 4.76	7.68 ± 5.45	−0.000	0.995
Interferon	2.31 ± 0.99	8.04 ± 4.42	8.12 ± 6.97	0.068	0.050
Ostilomavir	2.78 ± 9.22	8.02 ± 4.22	7.54 ± 5.33	−0.091	0.004^**^
β-Lactam/BLI	2.24 ± 0.77	7.79 ± 3.94	8.30 ± 8.24	0.054	0.055
Cephalosporine	2.46 ± 6.14	8.10 ± 4.57	8.38 ± 8.40	−0.008	0.771
Carbapenem	2.50 ± 6.77	8.16 ± 4.24	8.48 ± 7.85	−0.046	0.175
Aminoglycosides	2.46 ± 1.09	7.81 ± 3.23	8.38 ± 8.40	−0.002	0.945
Colistin	2.28 ± 0.72	8.18 ± 4.22	8.24 ± 7.52	−0.026	0.399
Ceftalazone-Avibactam	2.20 ± 0.61	9.40 ± 5.49	10.15 ± 10.56	−0.020	0.506
Ceftazidime-Tazobactam	2.18 ± 0.48	9.37 ± 4.48	9.26 ± 8.27	−0.012	0.667
Vancomycin	2.51 ± 7.21	8.15 ± 4.23	8.63 ± 7.84	−0.021	0.476
Linezolid	2.22 ± 0.72	8.30 ± 4.43	9.44 ± 8.91	−0.064	0.029^**^
Antifungal	2.22 ± 0.59	7.91 ± 4.30	8.31 ± 8.49	0.007	0.796

**Notes.**

NLR values represent baseline measurements at ICU admission (Day 1). Significance (*p*-value) reflects differences in baseline NLR between patients who received the specified treatment versus those who did not, derived from a Generalized Linear Model (GLM) adjusted for age, gender, and comorbidities. This analysis does not evaluate longitudinal NLR changes post-treatment.

** Shows statistical significance, *p* < 0.05.

**Table 3:** Mean survival time (days) across three treatments.

**Drug**	**Mean survival time (Days)**	**95% confidence interval for mean**	**Median survival time (Days)**	**95% Confidence interval for the median**
**Hydroxychloroquine**	54.385	48.848, 59.923	30.000	26.949, 33.051
**Chloroquine**	54.402	48.857, 59.948	29.000	25.999, 32.001
**Azithromycin**	54.864	49.331, 60.397	30.000	27.221, 32.779
**Kaletra (Lopinavir/Ritonavir)**	54.521	48.967, 60.075	30.000	27.146, 32.854
**Favipiravir**	54.232	48.703, 59.761	29.000	25.999, 32.001
**Remdesivir**	54.396	48.850, 59.942	29.000	26.000, 32.000
**Ribavirin**	54.417	48.870, 59.964	29.000	26.004, 31.996
**IVIG**	54.426	48.875, 59.977	29.000	25.954, 32.046
**Interferon**	54.394	48.848, 59.940	29.000	26.002, 31.998
**Ostilomavir (Tamiflu)**	57.911	51.451, 64.372	31.000	27.750, 34.250
**B-lactam/B-lactamase Inhibitors**	54.318	48.792, 59.845	29.000	25.986, 32.014
**Cephalosporins**	54.642	49.085, 60.198	30.000	26.936, 33.064
**Carbapenems**	54.465	48.919, 60.011	29.000	25.996, 32.004
**Aminoglycosides**	54.400	48.853, 59.948	29.000	26.005, 31.995
**Colistin**	54.365	48.831, 59.900	30.000	26.997, 33.003
**Ceftazidime-avibactam**	54.473	48.942, 60.004	30.000	26.995, 33.005
**Ceftazidime-tazobactam**	54.477	48.959, 59.994	30.000	27.026, 32.974
**Vancomycin**	54.467	48.925, 60.009	30.000	27.003, 32.997
**Linezolid**	55.072	49.282, 60.863	30.000	26.910, 33.090
**Tocilizumab**	60.813	51.792, 69.835	38.000	26.754, 49.246

**Figure 4:**
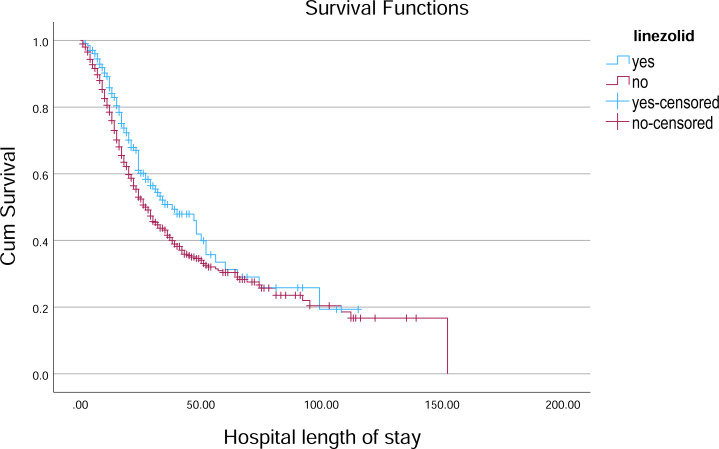
Kaplan–Meier survival curve for hospital length of stay by Linezolid. Kaplan–Meier survival curves illustrate the probability of remaining hospitalized over time, stratified by Linezolid treatment status.

**Figure 5:**
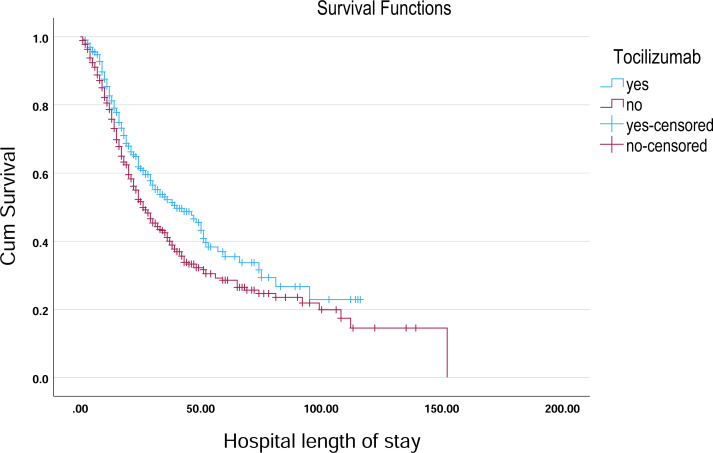
Kaplan–Meier survival curve for hospital length of stay by tocilizumab. Kaplan–Meier survival curves illustrate the probability of remaining hospitalized over time, stratified by tocilizumab treatment status.

**Figure 6:**
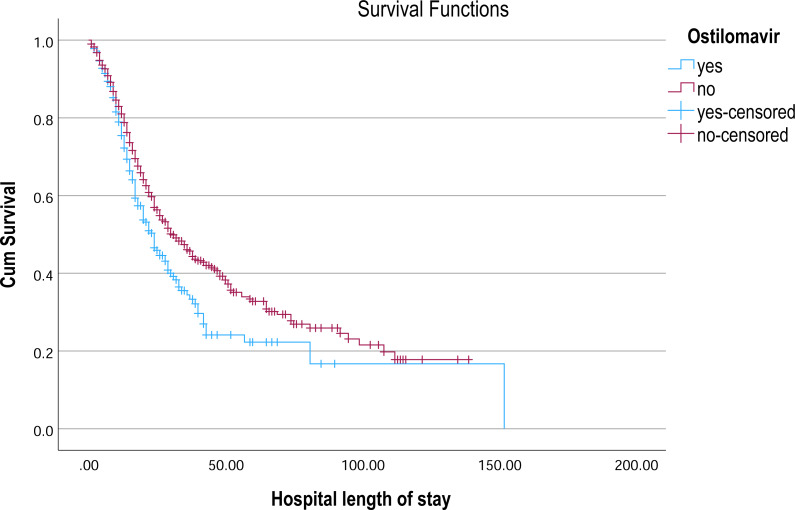
Kaplan–Meier survival curve for hospital length of stay by ostilomavir. Kaplan–Meier survival curves illustrate the probability of remaining hospitalized over time, stratified by oseltamivir treatment status.

### Survival of patients receiving different treatments

In the analysis of survival outcomes for various treatments, several drugs demonstrated similar survival times, with the majority of patients surviving around 29 to 30 days. The study showed that tocilizumab was associated with the highest mean survival time of 60.81 days (95% CI [51.79–69.83]) and a median survival of 38 days (95% CI [26.75–49.25]), showing a potential survival benefit in critically ill patients. Oseltamivir also showed a comparatively higher mean survival of 57.91 days (95% CI [51.45–64.37]) and a median of 31 days (95% CI [27.75–34.25]), indicating a modest advantage. Linezolid had a mean survival of 55.07 days (95% CI [49.28–60.86]) and a median of 30 days (95% CI [26.91–33.09]), aligning closely with general trends but still slightly above the average. In comparison, most other treatments, such as hydroxychloroquine (54.38 days; 95% CI [48.85–59.92]) and Favipiravir (54.23 days; 95% CI [48.70–59.76]), showed similar survival patterns with median survival times around 29–30 days (Table 3, Figs. 4–6).

## Discussion

This study provided a comprehensive review of 1,490 patients with COVID-19 who received nineteen different treatments from 15 tertiary public and private hospitals across KSA. The findings of this study provided valuable insights into the role of immune response markers, NLR, in predicting treatment outcomes for critically ill COVID-19 patients. The results highlighted that while numerous treatments were assessed, tocilizumab, ostilomavir, and linezolid demonstrated notable efficacy in modulating systemic inflammation and immune dysregulation, which are key factors driving disease severity in COVID-19. Tocilizumab, a monoclonal antibody targeting the IL-6 receptor, demonstrated a statistically significant reduction in NLR (*p* = 0.031), suggesting its effectiveness in mitigating the cytokine storm. This finding is consistent with a previous literature review which showed the role of IL-6 in driving systemic inflammation and poor outcomes in COVID-19 patients (Teachey et al., 2013; Qin et al., 2020; Tomita et al., 2011). Ostilomavir (Tamiflu) showed a significant reduction in NLR (*p* = 0.004), indicating its potential role in reducing systemic inflammation. This finding is particularly interesting given that ostilomavir is traditionally used as an antiviral agent rather than an anti-inflammatory drug. The weak positive correlation between ostilomavir and NLR (*r* = 0.077, *p* = 0.004) suggests that its anti-inflammatory effects may be secondary to its antiviral activity. However, the shorter mean survival time in patients treated with ostilomavir (40.635 days) compared to those not receiving the treatment (57.911 days) raises questions about its overall efficacy in improving patient outcomes. Despite the positive findings on the effectiveness of oseltamivir, several adverse reactions to oseltamivir have been reported, including nausea, vomiting, and rash (Strong et al., 2010). Linezolid demonstrated a statistically significant association with NLR. Crucially, linezolid is a broad-spectrum antibiotic targeting Gram-positive bacteria, and its use in this cohort was specifically for suspected or confirmed secondary bacterial pneumonia complicating severe COVID-19 (cite institutional protocols if possible, or general refs on co-infections). Its observed association with NLR is therefore highly likely to reflect either: (1) the baseline inflammatory burden associated with the bacterial co-infection itself, which prompted its administration, or (2) the subsequent modulation of inflammation resulting from effective treatment of the co-infection. It is essential to underscore that linezolid has no known direct antiviral activity against SARS-CoV-2 nor is it indicated for COVID-19 treatment per se; its use in this context remains off-label, targeting solely the bacterial complication (Strong et al., 2010; Mungmunpuntipantip & Wiwanitkit, 2021). While a small letter proposed theoretical exploration of linezolid for COVID-19 (Mungmunpuntipantip & Wiwanitkit, 2021), robust clinical evidence supporting its efficacy *against the virus* is lacking. Consequently, interpreting the NLR association with linezolid as evidence of anti-COVID-19 efficacy would be misleading. This finding instead highlights the impact of bacterial co-infection and its management on the inflammatory milieu in critically ill COVID-19 patients. Given the likelihood that Linezolid was administered in the context of co-infection, a variable not uniformly documented in this study, its observed impact on NLR may reflect underlying disease severity rather than a direct anti-inflammatory or immunomodulatory effect. The study strengthens the utility of NLR as a reliable biomarker for assessing systemic inflammation and predicting disease severity in COVID-19. Elevated NLR levels, as observed in this study (mean NLR = 8.77 ± 8.64), have been consistently linked to worse outcomes in respiratory illnesses, including COVID-19 (Forget et al., 2017; Tomita et al., 2011; Lee et al., 2010; Azab et al., 2012; Proctor et al., 2011; Koza, 2016; Hao et al., 2017; De Jager et al., 2012; Terradas et al., 2012; Bozbay et al., 2014). The significant effects of tocilizumab, oseltamivir, and linezolid on NLR further validate its role as a predictor of treatment efficacy. However, the weak correlations between NLR and specific treatments suggest that NLR alone may not be sufficient to guide therapeutic decisions. The findings of this study are consistent with previous research highlighting the importance of immune dysregulation and cytokine storms in COVID-19 pathogenesis (Teachey et al., 2013; Moore & June, 2020). The significant reduction in NLR with tocilizumab aligns with its established role in managing CRS and severe COVID-19 cases (Guaraldi et al., 2020; Buonacera et al., 2022; Sanders et al., 2020).

However, the lack of impact of hydroxychloroquine on NLR in our study aligns with subsequent large-scale randomized controlled trials and meta-analyses (Jarczak & Nierhaus, 2022) which conclusively demonstrated no clinical benefit for COVID-19 treatment and led to recommendations against its use. The limited impact observed for remdesivir and interferon on NLR is consistent with mixed or more nuanced results reported in the literature regarding their efficacy in modulating inflammation and improving outcomes in severe COVID-19 (World Health Organization, 2025).

This study reinforces the value of NLR as a responsive biomarker for treatment monitoring in severe COVID-19, showing associations with tocilizumab, oseltamivir, and linezolid. While NLR’s dynamic nature shows interpretive challenges, the findings support its clinical relevance. Rather than limiting the study, this highlights its strength in prompting deeper exploration into dynamic immune markers, encouraging a more comprehensive, multimodal approach to assessing therapeutic response.

## Limitations of the Study

Our study analyzed treatment effects on NLR at defined time points during ICU hospitalization rather than tracking daily drug administration. While study reports mean NLR values by treatment group, the exact duration of each drug therapy was not included as a separate variable in our analysis. Moreover, CRP and PaO2/FiO2 data were not systematically available across all 15 participating centers, precluding their inclusion. As demonstrated by Regolo et al. (2022), CRP and NLR provide complementary prognostic information in COVID-19, where CRP reflects acute-phase inflammation, and NLR measures cellular immune dysregulation through neutrophil-lymphocyte imbalance. Their combined assessment offers better risk stratification than either marker alone. Additionally, study (Regolo et al., 2023) found that high neutrophils, low lymphocytes, elevated NLR, and increased CRP are collectively associated with impaired oxygenation (PaO_2_/FiO_2_), a key factor in the severity of respiratory failure. Moreover, the study did not stratify patients by specific complications of COVID-19 (*e.g.*, bacterial pneumonia, thromboembolism, respiratory failure, or septic shock).

## Conclusions

This study evaluated the relationship between immune response markers, specifically NLR, and the efficacy of various treatments in patients with severe respiratory illnesses. Among the evaluated therapies, tocilizumab and oseltamivir showed a consistent trend of lower NLR values in both survivors and non-survivors, compared to those not receiving these treatments. Importantly, oseltamivir was associated with the lowest mean NLR in non-survivors, suggesting a potential role in reducing inflammatory burden and mortality risk.

In contrast, linezolid, used for bacterial co-infections, showed notable NLR variation. Its association with a higher mean NLR in non-survivors (10.126) likely signals the presence of more severe co-infections or a higher baseline inflammatory state in these patients, reinforcing NLR’s role as a biomarker of overall disease severity rather than indicating Linezolid’s efficacy against COVID-19 mortality. These findings suggest that tocilizumab and oseltamivir may offer some efficacy in modulating immune response (as measured by NLR) and potentially improving outcomes. However, due to observed weak correlations, no single therapy alone appears sufficient to predict or reduce mortality, emphasizing the need for multimodal treatment strategies and further investigation into combined biomarker models.

##  Supplemental Information

10.7717/peerj.20003/supp-1 Supplemental Information 1 Data and code

## Additional Information and Declarations

### Data Availability

The following information was supplied regarding data availability: The raw measurements are available in the Supplementary File.
